# Heme-mediated apoptosis and fusion damage in BeWo trophoblast cells

**DOI:** 10.1038/srep36193

**Published:** 2016-10-31

**Authors:** Mingli Liu, Salifu Hassana, Jonathan K. Stiles

**Affiliations:** 1 Department of Microbiology, Biochemistry and Immunology, Morehouse School of Medicine, Atlanta, Georgia 30310, United States of America

## Abstract

Placental malaria (PM) is a complication associated with malaria infection during pregnancy that often leads to abortion, premature delivery, intrauterine growth restriction and low birth weight. Increased levels of circulating free heme, a by-product of *Plasmodium*-damaged erythrocytes, is a major contributor to inflammation, tissue damage and loss of blood brain barrier integrity associated with fatal experimental cerebral malaria. However, the role of heme in PM remains unknown. Proliferation and apoptosis of trophoblasts and fusion of the mononucleated state to the syncytial state are of major importance to a successful pregnancy. In the present study, we examined the effects of heme on the viability and fusion of a trophoblast-derived cell line (BeWo). Results indicate that heme induces apoptosis in BeWo cells by activation of the STAT3/caspase-3/PARP signaling pathway. In the presence of forskolin, which triggers trophoblast fusion, heme inhibits BeWo cell fusion through activation of STAT3. Understanding the effects of free plasma heme in pregnant women either due to malaria, sickle cell disease or other hemolytic diseases, will enable identification of high-risk women and may lead to discovery of new drug targets against associated adverse pregnancy outcome.

Syncytial trophoblasts (ST) cover fetal villi and are responsible for transport of nutrients and exchange of waste between the mother and the developing fetus. Syncytiotrophoblast cells do not replicate but acquire fresh nuclei from underlying cell layer of mononucleated cytotrophoblasts. Fusion and conversion from the mononucleated to the syncytial state are mandatory for successful pregnancy, as the syncytiotrophoblast cells conduct endocrine function, produce key regulators for maintaining the pregnancy and supporting fetal growth in addition to exchange of gas and nutrients^1^. Differentiation of human placental villous trophoblast includes syncytial fusion of cytotrophoblast to form syncytiotrophoblast^2^. The morphologic and functional differentiation of human trophoblast cells culminates when the terminally differentiated multinucleated syncytial trophoblast is formed^2^,^3^. Placental malaria involves *P. falciparum* infected erythrocytes (pRBC) binding to trophoblasts, resulting in inflammation and pathology that is associated with poor pregnancy outcomes. The interaction between ST and pRBC is reminiscent of the interaction between pRBC and the blood brain barrier that exacerbates cerebral malaria (CM). We have previously shown that excess free heme, a product of hemolysis associated with erythrocyte damage due to malaria infection and/or hemoglobinopathies compromises the blood brain barrier (BBB) causing the barrier to become leaky and dysfunctional, thereby exacerbating CM complications. However, the effect of free heme on the integrity of the placental barrier and the effects of free heme on pregnancy outcome are unclear. Several studies reported the involvement of free heme in preeclampsia (PE), which described higher plasma^4^,^5^ and urine heme^5^ (both Hb-A and Hb-F) in women with PE compared to control. Since hemoglobin-induced oxidative stress is a pathogenic factor in preeclampsia, the radical scavenger and heme-binder α1-microglobulin (A1M) was significantly increased in plasma of women with PE^4^, while the human endogenous Hb-, and heme-scavenging systems such as haptoglobin (Hp) and hemopexin (Hpx) were significantly reduced. *In vitro*, the fusion of trophoblast-derived choriocarcinoma cell line BeWo can be triggered by forskolin (7 beta-acetoxy-1alpha, 6beta, 9alpha-trihydroxy-8,13-epoxy-labd-14-en-11-one), a pharmaceutical drug derived from the plant *Coleus forskohlii* (Lamiaceae), as a general direct, rapid and reversible activator of adenylyl cyclase^6^,^7^, recapitulating the *in vivo* process. Therefore in the present study we determined the effect and molecular mechanisms of heme on the syncytial fusion triggered by forskolin using the BeWo cell line, a trophoblast-derived cell line.

## Results

### Heme induces apoptosis in BeWo cells

**Figure 1:**
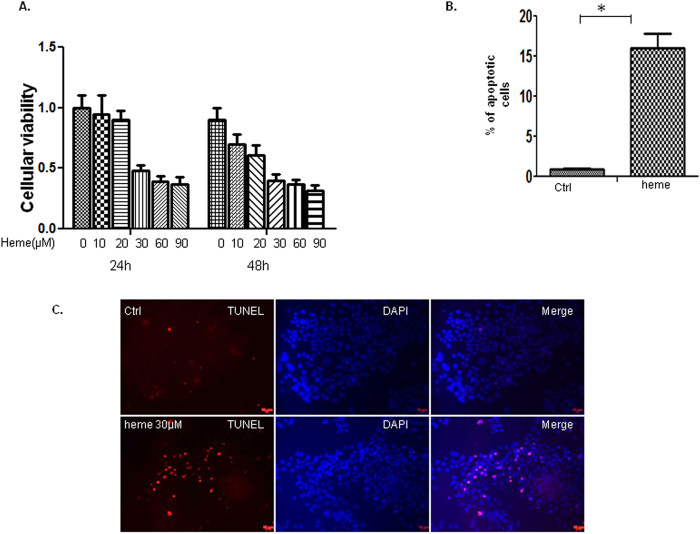
Heme induces BeWo cells apoptosis. BeWo cells were serum-starved for 24 h and then treated with 30 μM of heme for 24 h and 48 h respectively followed by MTT and TUNEL assay. (**A**). The viability of the untreated controls was defined as 1.0 in the MTT assays. Heme induced 5–63% and 10–68% of cell death when treated with 10 to 90 μM of heme for 24 h and 48 h accordingly with 30–90 μM causing maximum effects. (**B**,**C**). Cell death progression assayed by TUNEL were conducted, 10 fields were chosen randomly to count the TUNEL-positive cells in the slide using a 20 × microscope objective. Apoptotic indices (% of TUNEL-positive cells/total cell nuclei × 100) were calculated after counting cells. The apoptotic cells were found to be increased by heme treatment, therefore the reduced cell viability by heme was due to apoptosis.

Previous results showed that 30 μM heme induces human brain microvascular endothelial cell (HBVEC) apoptosis^8^ *in vitro*. HBVEC is a very important component of the blood brain barrier (BBB). The activation and disruption of HBVEC contributes to the pathogenesis of severe malaria such as cerebral malaria (CM). To test whether heme also induces apoptosis of BeWo cells, we examined the effects of heme on BeWo viability. Using the same procedure as described previously^8^, BeWo cells were serum-starved for 24 h and then treated with 30 μM of heme for 24 h and 48 h respectively, followed by MTT and TUNEL assay. The viability of the untreated controls was defined as 1.0 in the MTT assays. Heme induced 5–63% and 10–68% of cell death when treated with 10 to 90 μM of heme for 24 h and 48 h accordingly (Fig. 1A) with 30–90 μM causing maximum effects. Cell death progression was assayed by TUNEL which indicated that the reduced cell viability by heme was due to apoptosis (Fig. 1B,C). We randomly chose 10 fields to count the TUNEL-positive cells in the slide using a 20× microscope objective. Apoptotic indices (% of TUNEL-positive cells/total cell nuclei  × 100) were calculated after counting cells^9^,^10^. The apoptotic cells were found to be increased by heme treatment when assessed by TUNEL assay.

### Heme induces apoptosis in BeWo cells through activation of STAT3/caspase-3/PARP and P73 signaling pathways

**Figure 2:**
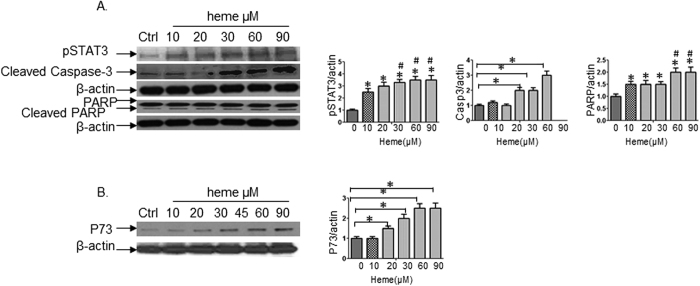
Heme induces apoptosis of BeWo cells through activation of STAT3/caspase-3/PARP and p73 signaling pathways. Cell lysates from BeWo cells treated with different concentration of heme as indicated were immunoblotted with anti-pSTAT3/STAT3, anti-cleaved caspase-3, anti-PARP and anti-p73 antibody. (**A**) The results showed that heme-induced STAT3 phosphorylation (pSTAT3) in BeWo *in vitro*, pSTAT3 exhibited a dose-dependent pattern when the cells exposed to increased heme concentration (*indicating the comparison of heme at different concentration to control, p < 0.05). The densitometric analysis further showed that the difference was significant between 10 μM of heme compared to 30 μM to 60 μM of heme (^#^indicating the comparison between high heme concentration and 10 μM of heme, p < 0.05). In addition, heme up-regulated the expression of caspase-3, cleaved caspase-3 increased in a dose-response manner. As cells exposed to heme treatment, PARP expression stay stable, however cleaved PARP was up-regulated which is in parallel with cleaved caspase-3 (*indicating the comparison of heme at different concentration to control, p < 0.05). The densitometric analysis further showed that the difference was significant between 10 μM of heme compared to 60 μM to 90 μM of heme (^#^indicating the comparison between high heme concentrations and 10 μM of heme, p < 0.05). (**B**) In BeWo cells, *p73* gene was involved in the apoptosis of trophoblasts induced by heme. Taken together, our results indicated that apoptosis of BeWo cells caused by heme was through the activation of STAT3/ caspase-3/PARP and p73 signaling pathways.

To define the signal transduction pathway utilized by heme to induce apoptosis, cell lysates from BeWo cells treated with different concentrations of heme were immunoblotted with anti-pSTAT3, anti-STAT3 and anti-β-actin antibody respectively. The results in Fig. 2A showed that heme-induced STAT3 phosphorylation (pSTAT3) in BeWo *in vitro.* pSTAT3 exhibited a dose-dependent pattern when the cells were exposed to increased heme concentration (*indicating the comparison of heme at different concentration to control, p < 0.05). The densitometric analysis further showed that the difference was significant between 10 μM of heme compared to 30 μM to 60 μM of heme (^#^indicating the comparison between high heme concentration and 10 μM of heme, p < 0.05). In the family of 13 aspartate-specific cysteine proteases, caspase-3 has the highest homology to the Ced-3 protease which is necessary for developmental cell death^11^ and plays central roles in the execution of apoptotic program^12^. Caspase-3 has been found to function downstream of activation of STAT3^12^,^13^. When cleaved caspase-3 expression was detected by Western blot analyses following stimulation with heme, cleaved caspase-3 was upregulated (Fig. 2A). Furthermore, cleaved caspase-3 increased in a dose-dependent manner in the dose-course experiments. Caspase-3 is primarily responsible for the cleavage of poly(ADP-ribose) polymerase (PARP)^14^. PARP is a nuclear enzyme that contains two zinc finger domains near its amino terminus and serves as a substrate for caspase-3^11^, which is a more broader apoptotic marker than caspase-3 itself. In our experiments, as cells were exposed to heme treatment, PARP expression stayed stable. However, cleaved PARP was up-regulated in parallel with expression of cleaved caspase-3. There were significant differences in cleaved PARP between low and high heme concentration (Fig. 2A, *indicating the comparison of heme at different concentration to control, p < 0.05). The densitometric analysis further showed that the difference was significant between 10 μM of heme compared to 60 μM to 90 μM of heme (^#^indicating the comparison between high heme concentrations and 10 μM of heme, p < 0.05). We recently found that expressions of several genes were altered when HBVECs underwent apoptosis induced by hemestimulation. Among these genes, tumor protein p73 was one of the significant mediators of the apoptotic effects^15^. In BeWo cells, we observed a similar p*73* gene regulated apoptosis of trophoblasts induced by heme (Fig. 2B). Taken together, our results indicate that apoptosis of BeWo cells caused by heme was mediated through the activation of STAT3/caspase-3/PARP and p73 signaling pathways.

### BeWo cell fusion is reduced in the presence of heme

**Figure 3:**
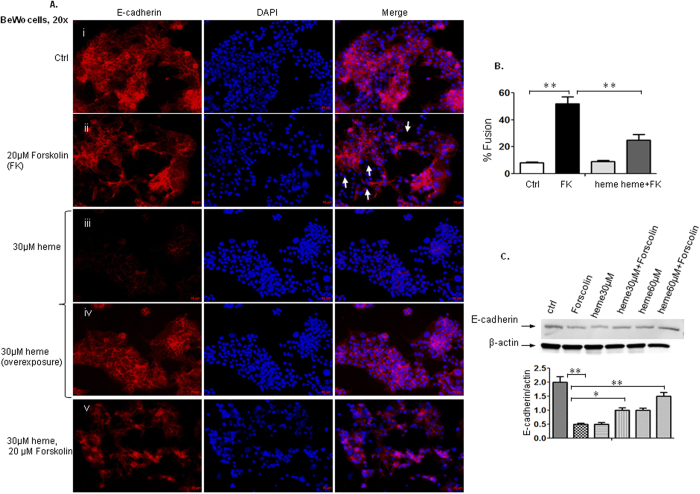
BeWo cell fusion is reduced in the presence of heme by detecting E-cadherin expression. The intercellular fusion of BeWo- a trophoblast-derived cell line, was examined by the rearrangement of E-cadherin (**A**,**B**). There was a low level of spontaneous fusion of BeWo cells, but most cells were in mononucleated state (**A**-i). When BeWo cells were treated with forskolin, cell borders and the E-cadherin staining vanished in fusing cells (**A**-ii, arrows). The labeling experiments were also used to quantify BeWo cell fusion under the different stimulatory conditions. Forskolin (FK) triggered a fusion rate of 52% (**A**,**B**) in BeWo cells after 48 h cultivation. Heme decreased the expression of E-cadherin (**A**-iii, while A-ivis an overexposed image of **A**-iii). Immunofluorescence staining of BeWo cells in the presence of 30 μM heme in combination with20 μM forskolin showed that forskolin-induced cell fusion was inhibited by heme (**A**-v,**B**), the fusion rated decreased to 25%. (**C**). The immunoblot approach was used to determine whether heme may affect E-cadherin expression under various stimuli as well. The expression of E-cadherin was suppressed by forskolin compared to control, while enhanced in the presence of heme in combination with forskolin compared to forskolin only. The immunoblot data were thus consistent with the immunofluorescence data.

Since fusion and changes from the mononucleated to the syncytial state are critical for a successful pregnancy, we next focused on intercellular fusion of BeWO, a trophoblast-derived cell line, as indicated by the rearrangement of E-cadherin. In untreated trophoblast-derived BeWo cells serving as the control, there was a low level of spontaneous fusion similar to that reported by others^16^ although most cells were in the mononucleated state (Fig. 3A-i). Forskolin stimulated fusion of BeWo cells by up-regulating intracellular cAMP levels through adenyl cyclase activation^1^,^17^. Morphologically, as cells fused to form multinucleated syncytia, E-cadherin immunofluorescence staining was markedly reduced, being present only at points of cellular contact between unfused mononuclear cells^3^. Thus induce dcell fusion was assessed by the loss of E-cadherin expression using immunofluorescence staining. When BeWo cells were treated with forskolin, cell borders and the E-cadherin staining disappeared in fusing cells (Fig. 3A-ii, arrows). In Fig. 3Aii the areas indicated by white arrows show the presence of nucleus but the absence of cell bordering, which is a representation of cells fused by forskolin treatment. The labeling experiments were also used to quantify BeWo cell fusion under different stimulatory conditions. By carefully quantifying a total of at least 20 randomly selected microscopic fields of each condition, the number of nuclei in syncytia and total number of nuclei were calculated and the ratios of number of nuclei in syncytia/total number of nuclei were referred to as fusion index. Forskolin triggered a fusion rate of 52% (Fig. 3A-ii, B) in BeWo cells after 48 h cultivation. As observed, heme decreased the expression of E-cadherin (Fig. 3A-iii, A-iv is an overexposed image of Fig. 3A-iii). E-cadherin was detected at much lower levels after treatment with heme, which indicates that E-cadherin expression was reduced by heme (Fig. 3A-iii; confirmed by Western blot, Fig. 3C). To compare fusion induced by forskolin to that of heme, we overexposed image as shown in Fig. 3A-iv, which showed that heme per se did not significantly affect fusion of BeWo cells. The reason why heme decreased the expression of E-cadherin is beyond the scope of this manuscript. However, we plan to investigate this mechanism further in the next manuscript. Immunofluorescence staining of BeWo cells in the presence of 30 μM heme in combination with 20 μM forskolin showed that forskolin-induced cell fusion was inhibited by heme (Fig. 3A-v,B) to 25%. We also used an immunoblot approach to determine whether heme may affect E-cadherin expression under various stimuli. The expression of E-cadherin was suppressed by forskolin compared to control, while enhanced in the presence of heme in combination with forskolin when compared to forskolin only (Fig. 3C). Thus, the immunoblot data were consistent with the immunofluorescence data.

**Figure 4:**
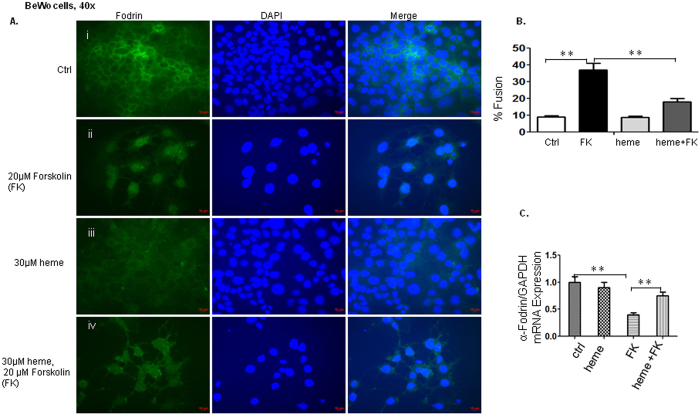
BeWo cell fusion is reduced in the presence of heme by detecting α-fodrin expression. The similar effects were observed in trophoblast cells when conversion from the mononucleated to the syncytial state occurred by detecting α-fodrin expression. (**A**) The BeWo cells showed apparent α-fodrin staining, the cells were mostly in the mononucleated stages when there was lack of stimulation (**A**-i). Treatment of forskolin increased differentiation and fusion of BeWo cells visualized by absence of α-fodrin staining (**A**-ii) demonstrating syncytialisation. In the presence of heme, more individual BeWo cells stained positive for α-fodrin (**A**-iv). (**B**). The immunofluorescence assays were quantified and reported as percentage of nuclei in syncytia (%fusion). While controls showed a spontaneous fusion rate of about 9%, forskolin treatment enhanced fusion events significantly to 37%. The fusion rate was reduced to 18% in the presence of 30 μM heme combined with forkolin compared to that of forskolin only. (**C**). The results revealed that down-regulation of α-fodrin mRNA expression was pronounced in BeWo cells treated with forskolin. Stimulation with this fusion- inducing reagent led the α-fodrin mRNA expression level down to 40% compared with control after 48 h cultivation. There was a significant increase towards a much higher expression of α-fodrin mRNA in the presence of heme and forskolin compared with that in the presence of forskolin only, which indicate reduced fusion. The results support the BeWo cell fusion is reduced in the presence of heme.

We also examined cell-cell fusion in response to forskolin and heme by focusing on the remodeling of spectrin-α-fodrin, a major component of the sub-α-membranous cytoskeleton^1^,^18^. Similar effects were observed in trophoblast cells when conversion from the mononucleated to the syncytial state occurred. The BeWo cells showed apparent α-fodrin staining when the cells were mostly in the mononucleated stages and were in the absence of stimulation (Fig. 4A-i). Treatment of forskolin increased differentiation and fusion of BeWo cells visualized by the absence of α-fodrin staining (Fig. 4A-ii) demonstrating syncytialisation. In the presence of heme along with forskolin, more individual BeWo cells positively stained with α-fodrin (Fig. 4A-iv) indicating reduced fusion rate. Further quantification of BeWo cell fusion confirmed the role of forskolin (trigger cell fusion) and heme in these cells: while controls showed a spontaneous fusion rate of about 9%, forskolin treatment enhanced fusion events significantly to 37%, whereas the fusion rate was reduced to 18% in the presence of 30 μM heme and forskolin compared with that of forskolin only (Fig. 4B). The results in Fig. 4C revealed that down-regulation of α-fodrin mRNA expression was pronounced in BeWo cells treated with forskolin, as this fusion- inducing reagent led the α-fodrin mRNA expression level down to 40% compared with control after 48 h cultivation. On the contrary, there was a significant increase towards a much higher expression of α-fodrin mRNA in the presence of heme and forskolin compared with that of forskolin only. The results are in accordance with immunostaining data and support that BeWo cell fusion is reduced in the presence of heme.

### Heme reduces mRNA expression of cell-fusion related genes when forskolin induces syncytialation

**Figure 5:**
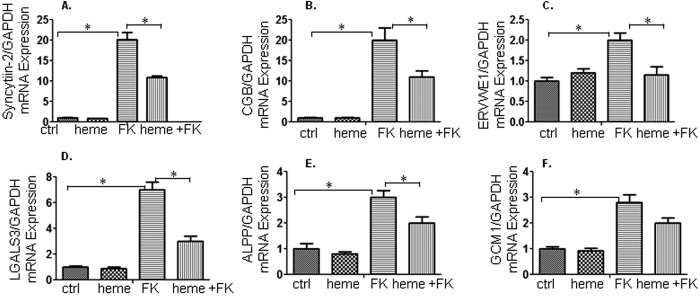
Heme reduces mRNA expressions of cell-fusion related genes of syncytialisation induced by forskolin. We utilized quantitative PCR (real-time PCR) to determine changes in the mRNA levels following each treatment as indicated. (**A**) We found that forskolin led to an increase in mRNA for Syncytin-2, while heme inhibited the Syncytin-2 expression induced by forskolin. (**B**–**F**) Furthermore, the mRNA expressions of commonly used markers for syncytialisation *CGB, ERVWE1, GCM1, ALPP* and *LGALS3* were assessed in the presence of forskolin or heme in combination with forskolin. In the presence of forskolin, the mRNA expression of *CGB, ERVWE1*,*GCM1, ALPP* and *LGALS3* were significantly up-regulated compared with the controls. Addition of heme to the culture medium in the presence of forskolin led to a significant decrease of *CGB, ERVWE1, ALPP* and *LGALS3* compared with that in the presence of forskolin only. The mRNA expression of *GCM1* was not significantly changed in the presence of heme and forskolin compared with that in the presence of forskolin only, although there was a trend towards the expression level of control.

After establishing that forskolin-triggered cell fusion was reduced by heme, we proceeded to determine whether the trophoblast fusion-markers were down-regulated accordingly following treatment with heme. We utilized quantitative PCR (real-time PCR) to determine changes in mRNA levels following the different treatments. Syncytin-2 is encoded by the envelop gene of endogenous retrovirus-FRD (ERVFRD-1) and plays a critical role in fusion of placental trophoblasts resulting in the formation of the multinucleated syncytiotrophoblast^19^. We observed that forskolin treatment increased Syncytin-2 mRNA levels, while heme inhibited the Syncytin-2 mRNA expression induced by forskolin (Fig. 5A). Furthermore, the mRNA expression of commonly used markers for syncytialisation such ashuman chorionic gonadotropin (CGB), Syncytin-1 (ERVWE1), glial cells missing 1 (GCM1), human placental alkaline phosphatase (ALPP) and placental protein 13 (PP13 or LGALS3) were assessed in the forskolin or heme and forskolin combination treatments by real-time RT-PCR (Fig. 5B–F). In the presence of forskolin, the mRNA expression of CGB, ERVWE1, GCM1, ALPP and LGALS3 were significantly up-regulated compared with the controls. Addition of heme to the culture medium in the presence of forskolin led to a significant decrease of CGB, ERVWE1, ALPP and LGALS3 compared with those in the presence of forskolin only. The mRNA expression of GCM1 was not significantly changed in the presence of heme and forskolin compared with that in the presence of forskolin only, although there was a trend towards the expression level of control.

### Heme inhibits differentiation and fusion of BeWo cells through activation of STAT3 signaling pathways

**Figure 6:**
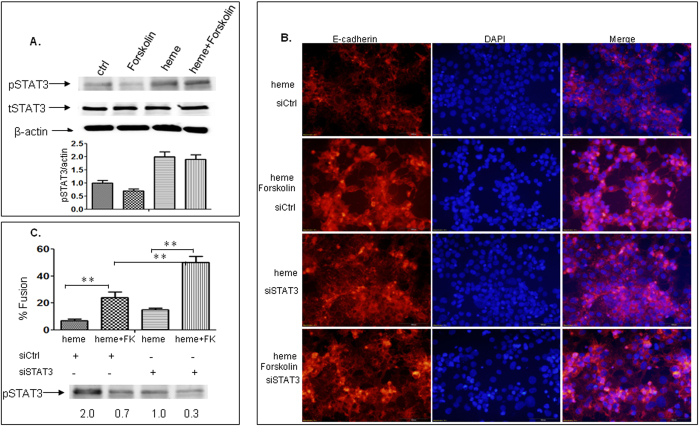
Heme inhibits differentiation and fusion of BeWo cells through activation of STAT3 signaling pathways. We monitored STAT3 alteration over time following stimulation of BeWo cells with heme, forskolin, or forskolin combined with heme by immunobloting. (**A**). We found that heme treatment led to a robust activation of STAT3 as described before. On the other hand, incubation with forskolin for 48 h led to deactivation of STAT3. The combination of heme and forskolin produced higher activated STAT3 than that in the presence of forskolin. (**B**,**C**). We next suppressed activation of STAT3 by introducing siSTAT3 into cell culture medium followed by stimulation of cells with heme, forskolin, or forskolin combined with heme and then cells were subjected to immunofluorescence staining. When STAT3 was inactivated, there was a significant increase towards a much higher fusion rate in the presence of heme and forskolin, which doubled that without siSTAT3 knock-down. Taken together, these results indicated that STAT3 activation mediated inhibition of BeWo fusion caused by heme.

SinceSTAT3 is activated when BeWo cells undergoes apoptosis, we also monitored STAT3 alteration over time following stimulation of BeWo cells with heme, forskolin, or forskolin combined with heme utilizing immunoblotting techniques. We found that heme treatment led to a robust activation of STAT3 as described before. On the other hand, incubation with forskolin for 48 h led to a significant decrease in activation of STAT3. The combination of heme and forskolin produced higher activated STAT3 than that in the presence of forskolin (Fig. 6A). These results indicate that activated STAT3 may play a role in heme-mediated inhibition of BeWo fusion. We next suppressed activation of STAT3 by introducing siSTAT3 into cell culture medium followed by stimulation of cells with heme, forskolin, or forskolin combined with heme, and then cells were subjected to immunofluorescence staining. When STAT3 was inactivated, there was a significant increase towards a much higher fusion rate in the presence of heme combined with forskolin which doubled that without siSTAT3 knock-down (Fig. 6B,C). Taken together, these results indicated that heme reduces ST integrity and that STAT3 activation mediates inhibition of BeWo fusion caused by heme.

### Malaria pigment hemozoin impairs BeWo cell fusion

**Figure 7:**
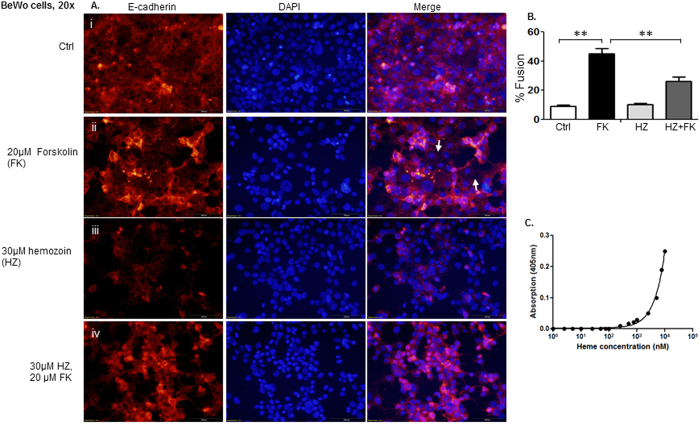
Malaria pigment hemozoin impairs BeWo cell fusion. We isolated hemozoin from parasite infected mouse liver tissue, followed by the examination of the effect of hemozoin on the fusion of BeWo cells. 30 μM of hemozoin (HZ) was chosen based on our previous data. Panel A: (i) BeWo cells were in mononucleated state. (ii) BeWo cell borders indicated by the E-cadherin staining vanished in fusing cells (white arrows) when cells were treated with forskolin. (iii) Hemozoin reduced the expression of E-cadherin. (iv) Hemozoin impaired the forskolin-induced BeWo cell fusion (Fig. 7A-ii vs. Fig. 7A-iv). Panel B: Cell fusion assay: Number of nuclei in syncytia and total number of nuclei were counted, and fusion index was calculated (number of nuclei in syncytial/total number of nuclei × 100). Panel C: Hemin (heme) quantification by a colorimetric technique- a sigmoid relationship was achieved between concentration and the blank-subtracted absorbance at 405 nm.

In order to determine whether parasite-derived hemozoin (HZ) contributes to the aforementioned placental events, we isolated hemozoin from parasite infected mouse liver tissue as previously described (Deroost *et al*.^20^), followed by the examination of the effect of hemozoin on the fusion of BeWo cells. 30 μM of hemozoin, which is equivalent to 30 μM hemin was chosen based on our previous data. From Fig. 7, we observed that hemozoin impaired forskolin- induced BeWo cell fusion in a way similar to that of hemin (Fig. 7A-ii vs. Fig. 7A-iv). Immunofluorescence staining of BeWo cells in the presence of hemozoin in combination with 20 μM forskolin showed that forskolin-induced cell fusion was inhibited by hemozoin from 45% to 26% (Fig. 7B). The unknown hemozoin concentration was calculated from the calibration curve of heme concentration (nM) versus absorbance at 405 nm. Fig. 7C: Hemin (heme) was quantified by a colorimetric technique- a sigmoid relationship was achieved between concentration and the blank-subtracted absorbance at 405 nm.

## Discussion

Placental malaria (PM) or pregnancy-associated malaria is characterized by many pathological alterations, such as sequestration of parasitized red blood cells (pRBC), infiltration of monocytes within the intervillous spaces of the placenta, cytotrophoblastic and syncyiotrophoblastic damage^21^,^22^,^23^,^24^ that disrupts the materno-fetal exchange system and leads to intra-uterine growth retardation and low birth weight^24^,^25^. Beyond malaria infection, local inflammation-triggered fetal growth restriction was found in a cohort of Malawian women and their infants^26^. Furthermore the association between local inflammation and micronutrient transport was confirmed in an *in vitro* model of placental malaria with local inflammation. Specifically maternal monocyte products impaired the activity of amino acid transporters on placental cells, leading to fetal growth restriction^26^. We have recently reported that free heme and heme-mediated signaling pathways play important roles in the pathogenesis of severe *P. falciparum* malaria (children/adults)^27^; and that elevated levels of IL-10 in maternal peripheral blood can serve as a biomarker associated with asymptomatic malaria in pregnant women^28^. This implies that the susceptibility to symptomatic malaria in pregnancy may be associated with high levels of IL-10. In addition, IL-10 also functions as a biomarker for inflammatory PM reported by Kabyemela *et al*.^29^. Furthermore, we have recently determined that women with hemoglobinopathies, such as sickle cell anemia (SCA) in West Africa, were at a greater risk of morbidity and mortality during pregnancy compared with women without hemoglobinopathies^30^. Women with sickle cell trait (AS) are at a higher risk of poor pregnancy outcomes than women with hemoglobin (Hb) AA status^31^,^32^,^33^. A common observation among malaria infected individuals and those with hemoglobinopathies is the elevation of free heme in their peripheral blood^34^. If this is indeed the case, improved pregnancy outcomes in women with PAM and SCA may be achieved through identification of novel predictive biomarkers related to heme and development of interventions targeting factors such as heme which mediates adverse pregnancy outcomes. Whether heme causes damage to the syncytial trophoblasts (ST) layer and crosses the placental barrier to harm the fetus, or in some other way compromises the intrauterine environment, has not been investigated. Proliferation, differentiation and apoptosis are hallmarks of the life cycle of the villous trophoblast. Maintenance of trophoblast turnover at equilibrium is mandatory for placental function. The balance between trophoblast apoptosis and proliferation represents a mechanism to control normal trophoblast invasion^35^. Although apoptosis is an essential process for normal placental development, it is highly increased in placental diseases^36^. An increase in trophoblast cell apoptosis may cause placental dysfunction^37^ via several reported mechanisms. Sharma *et al*.^24^ found that malaria infection in the placenta enhances oxidative stress, which in turn activates mitochondrial pathway of apoptosis (intrinsic pathway) at the materno-fetal exchange system and results in placental damage and insufficiency. Using BeWo cells, a widely used model of villous trophoblast cells and primary trophoblast cells, Stubert *et al*. observed that the pro-inflammatory cytokine tumor necrosis factor (TNF)-α exerts the pro-apoptotic effects on trophoblasts^37^. Furthermore, characterization of the apoptotic pathways indicated the specific action of calpain, an apoptosis inducing factor (AIF) pathway, in trophoblast apoptosis^38^. The reduced syncytin-1 level activatescalpain1 (the cysteine protease) which cleaves the AIF, leading to AIF nuclear translocation and triggering the apoptotic program. Another *in vitro* study revealed that the late phase of apoptosis of BeWo cells and human placental explants were triggered by serum deprivation. While caspase-3 and poly(ADP-ribose) polymerase-1 (PARP-1) are sequentially activated, leptin is capable of interrupting the pathway and promoting proliferation and survival of trophoblastic cells^39^. In addition, recent studies demonstrated ectopic expression and secretion of insulin growth factor (IGF-1)stimulated proliferation and reduced apoptosis in a mouse model of placental insufficiency *in vivo* and in the human trophoblast line BeWo *in vitro*^40^.

Since apoptosis plays a central role in placental physiology, we determined the effects of heme on the BeWo cell line, a trophoblast-derived cell line which maintains many characteristics of human trophoblast cells and has been widely used to study placental function^41^. Our results showed that heme induced apoptosis in BeWo cells via the activation of STAT3, caspase-3 and its substrates PARP. PARP cleavage is catalyzed by activated caspase-3 which prevents PARP-mediated DNA repair processes^39^. On the other hand *p73* gene was also activated. Trophoblast apoptosis, similar to other types of cell apoptosis, is mediated by extrinsic and intrinsic pathways, however, the pathway(s) activated by heme during trophoblast apoptosis remains to be determined.

During normal pregnancy, trophoblast apoptosis increases while placenta grows. As mononucleated villous trophoblast cells proliferate, some of them differentiate and fuse to form the syncytiotrophoblast^42^. Fusion of cytotrophoblasts with the overlying syncytiotrophoblast is an integral step in differentiation of the human placental villous trophoblast^1^. Gauster *et al*. revealed that caspase-dependent fragmentation of α-fodrin may be important for reorganization of the sub-membranous cytoskeleton during trophoblast fusion^1^. Probably, caspase-8-mediated extrinsic pathway is a prerequisite for differentiation and syncytial fusion of cytotrophoblast cells^2^. The results from Robinson’s group demonstrated that trophoblast cell fusion and differentiation are mediated by both the protein kinase C and A pathways^18^,^43^. Furthermore, recent studies uncovered Syncytin-2 is a downstream target of PKA. They showed that fusion-inducing factors such as forskolin, soluble cAMP, CREB2 and JunD need a CRE/AP-1-like motif to induce Syncytin-2 expression which leads to the formation of the peripheral syncytiotrophoblast layer^19^. In addition to Syncytin-2, the modulation of cell surface expression of its receptor and amino acid transport system B^0^ also were associated with syncytialization^44^.

In the present study, we detected E-cadherin and α-fodrin expression by immunofluorescent microscopy to represent fusion of trophoblast-BeWo cells. E-cadherin has been showed on the surface of cytotrophoblasts *in situ*, but not on the surface of the encompassing syncytiotrophoblasts^45^, indicating that E-cadherin expression was localized on the cell surface at points of cell-cell contact and could not be detected following cellular fusion^3^. E-cadherin is present in a dynamic state in cytotrophoblasts and is down-regulated as cellular fusion progressed^3^. This mechanism was elaborated in an *in vitro* experiment where E-cadherin turnover was mediated through cleavage of its extracellular domain in the culture medium^46^. Similar mechanisms have been proposed previously for the turnover of N-cadherin in lens cells^47^ and the fusion of myoblasts during myotube formation^48^,^49^. Like E-cadherin, α-fodrin in term placenta, was associated with apical and lateral cell borders of cytotrophoblasts but was absent in the overlying syncytiotrophoblasts^1^. Fragmentation of α-fodrin induced by caspase may be important for reorganization of the sub-membranous cytoskeleton during trophoblast fusion^1^. This is in accordance with early findings which demonstrated that fragmentation of α-fodrin occurs during differentiation of nerve cells^50^, lens fibres^51^,^52^, and during myoblast fusion^53^.

*Plasmodium* parasites produce a pigment, hemozoin to detoxify the free heme that is generated by hemoglobin degradation^54^. Some studies suggest that hemozoin is a pro-inflammatory factor, although others indicate that hemozoin may be beneficial to the host depending on the stage of infection. Hemozoin has recently been found to be a danger signal that results in the activation of the host inflammasome^55^. In order to determine whether parasite-derived hemozoin could contribute to the placental events, we isolated hemozoin from *Plamodium* infected mouse liver tissue, and examined its effect on the fusion of BeWo cells. As expected we observed similar effect on BeWo cell fusion as observed for hemin indicating that hemozoin impairs forskolin- induced BeWo cell fusion (Fig. 7A-ii vs. Fig. 7A-iv).

Many reports have challenged the paradigm that *Plasmodium vivax* malaria is a benign infection^56^. Low birth weight, abortion, premature delivery and intrauterine growth restriction are generally found in pregnant women infected by *P. vivax* malaria. Contrary to *Plasmodium falciparum* which shows the presence of hemozoin deposition and accumulation of inflammatory cells, *P. vivax*, the most widespread of the human *Plasmodium* species, is not generally found sequestered in placenta and is weakly bound to placental CSA^57^. Additionally, no hemozoin in macrophages or increased intervillous inflammatory cells are found^57^. Free heme could therefore be responsible for the promotion of apoptosis and the lack of fusion events within the placenta of *vivax* malaria pregnant women without attaching to trophoblasts. Future experiments with both human *Plasmodium* species would be needed to explore this phenomenon in the field.

BeWo cells have been widely used as *in vitro* models for studying placental uptake of a variety of nutrients including glucose^58^, amino acids^59^,^60^,^61^ and iron^62^ seeded onto permeable membrane supports. Under these conditions, the integrity of the cell layer is critical. BeWo cells do not undergo contact inhibition of growth making it more difficult to achieve and maintain an intact cell monolayer that would be ideal for transport studies. Some authors worked with cell multilayers to avoid risking the presence of gaps in a cell monolayer^62^. The presence of multilayered cellular structures may slow nutrient transport when compared to that of a monolayer. The cell density may not affect cell apoptosis if the cells are seeded constantly at specific densities with appropriate controls. It is unlikely that a slower transfer rate in this *in vitro* model will affect the fusion-apoptosis turnover.

In summary, we identified a new causative agent of inhibition of trophoblast fusion; heme. In the absence of forskolin, heme induced apoptosis in BeWo cells via activation of STAT3/caspase-3/ PARP while in the presence of forskolin, pSTAT3 was induce by heme to inhibit BeWo cell fusion. Our studies indicated that heme and its induced pathways are potential drug targets in the prevention of heme induced trophoblast cell apoptosis and fusion damage.

## Methods

### Antibodies and reagents

Rabbit antibody STAT3, phospho-STAT3, cleaved caspase-3, rabbit E-cadherin, and rabbit PARP were purchased from Cell Signaling Technology Inc. (Danvers, MA). Forskolin and polyclonal antibody to β-actin were obtained from Sigma-Aldrich (St. Louis, Mo). Mouse monoclonal antibody against α-fodrin was purchased from ABCAM (Cambridge, MA). All secondary antibodies used for Western blot were purchased from Calbiochem (La Jolla, CA). STAT3 siRNA, control siRNA and rabbit polyclonal antibody against p73 were purchased from Santa Cruz (Dallas, TX). Hemin (heme) was purchased from Frontier Scientific (Logan, UT).

### Culture of BeWo cells

BeWo cells were purchased from American Type Culture Collection (ATCC) and cultured according to the supplier’s instructions. In brief, cells were cultured in Kaighn’s modification of Ham’s F-12 with L-Glutamine, supplemented with 10% FBS, penicillin/streptomycin. Cells between passages 10 to 20 were used for *in vitro* experiments. Syncytialisation of BeWo cells was induced with forskolin as previously described^17^.

### Measurement of cell viability by MTT assay

BeWo cells were seeded at 1 × 10^4^ cells in 100 μl of medium per well into 96-well plates and serum-starved for 24 h, followed by exposing to heme at 10, 20, 30, 60 and 90 μM for 24 h. MTT assay was performed in accordance to the manufacturer’s instructions. 10 μl of MTT reagent (the ratio of MTT reagent to medium is 1:10) was added into each well and incubated in the dark at room temperature for 2 to 4 h. Absorbance at 570 nm was measured using 650 nm as reference filter by a CytoFluorTM 2300 plate reader and the software CytoFluorTM 2300 v. 3A1 (Millipore Co, Bedford, MA, USA).

### Immunofluorescence staining

Cells grown in monolayer cultures were fixed with 4% paraformaldehyde in phosphate-buffered saline (PBS), permeabilized with 0.2% Triton X-100, and blocked with 10% goat serum prior to antibody staining. Specific primary antibodies were added at 1:200 dilution overnight. Fluorescent staining was developed using the Alexa-488 or Alexa 555 fluorescence system (Molecular Probes, Carlsbad, CA). TUNEL assay, the *in situ* cell death detection kit (TMR red; Boehringer-Mannheim, Mannheim, Germany) was used. The sections were incubated with the TUNEL reaction solution for 60 min at 37 °C in the dark. Cover slips were mounted onto slides with Vectashield mounting medium with DAPI (H-1200; Vector Laboratories Inc). Fluorescent images were collected by using a Zeiss laser scanning microscope (LSM) 510 confocal microscope, and images were captured with LSM software, version 2.3 (Carl Zeiss, Wetzlar, Germany). Apoptotic cells and total cells were counted in ten randomly chosen microscopic fields, and the percentage of apoptotic cells was calculated and compared between the experimental and control groups.

### Cell fusion assay

BeWo cell fusion was assayed by methods previously described^43^. Briefly, BeWo cells were double stained for E-cadherin and nuclei (DAPI) to visualize cell borders and the number of nuclei per cell. Quantification were performed in a total of at least 20 randomly selected microscopic fields of each condition, in three independent experiments. Number of nuclei in syncytia and total number of nuclei were counted, and fusion index was calculated (number of nuclei in syncytial/total number of nuclei × 100).

### Real-time RT-PCR analysis

Cell pellets were stored in Trizol reagent and homogenized in fresh Trizol. Total RNA were isolated from cells using an RNeasy Mini Kit (Qiagen, Valencia, CA). Total RNA was quantified using the Nanodrop N-1000 byAgilent Biosystems (Santa Clara, CA). cDNA were synthesized from the isolated RNA using iScript cDNA Synthesis Kit (Bio-Rad Laboratories, Inc). Reverse transcription was performed by using random hexamers at 25 °C for 5 minutes, 42 °C for 30 minutes, and 85 °C for 5 minutes. Quantitative PCR were performed using iQ SYBR Green Supermix (Bio-Rad Laboratories, Inc.) in a CFX96 Real-Time PCR System machine (Bio-Rad Laboratories, Inc.). The data was analyzed using CFX96 Real-Time PCR System (Bio-Rad Laboratories, Inc). Primer sequences for the genes were described as previously and summarized in Table 1.

### Western blot

Cells were lysed with lysis buffer (50 mmHEPES, 150 mmNaCl, 1.5 mm MgCl2, 1 mmEGTA, 10% glycerol, 1% Nonidet P-40, 100 mm NaF, 10 mm sodium pyrophosphate, 0.2 mm sodium orthovanadate, 1 mm phenylmethylsulfonyl fluoride, 10 μg/ml aproptinin, and 10 μg/ml leupeptin). Samples were separated by SDS/PAGE, and separated proteins were transferred to nitrocellulose membranes and identified by immunoblotting. Primary antibodies were obtained from commercial sources, these antibodies were diluted at the ratio of 1:1000 according to manufacturer’s instruction, while secondary antibodies included HRP-conjugated anti-rabbit and anti-mouse antibodies were obtained from Calbiochem. Blots were developed with Supersignal Pico or Femto substrate (Pierce). A densitometric analysis of the bands was performed with the ImageQuant program (Bio-Rad).

### Hemozoin (HZ) determination in tissues

Extraction of hemozoin from perfused mouse tissues was performed as described previously^20^,^63^. Briefly, approximately 60 mg liver was homogenized with a solution containing 50 mM Tris/HCl pH 8.0, 5 mM CaCl_2_, 50 mM NaCl and 1% Triton X-100. The homogenate was supplemented with 1% Proteinase K and incubated overnight at 37 °C. The next day, the proteinase K digest was sonicated for 1 min (10 W, pulse 0.5 sec) and centrifuged at 11,000× g for 45 min. The supernatant was discarded and the pellet was washed three times in 100 mM NaHCO_3_, pH 9.0 and 2% SDS with subsequent sonication and centrifugation for 30 min to remove degraded tissue, free heme and Hb. After the third wash, the pellet HZ was dissolved and sonicated in 100 mM NaOH, 2% SDS and 3 mM EDTA to form hematin and centrifuged to pellet any remaining insoluble material. Finally, an aliquot was diluted in 1 M NaOH to determine the heme content by spectrophotometry (λ = 405 nm). The concentration of HZ was calculated by comparison to a standard curve of hemin (Fig. 7C) that was analyzed in the same way.

### Statistical analysis

Statistically significant differences were determined using Prism statistical software (Graph Prism 4.03, San Diego, CA). All data were presented as mean ± SEM of at least three independent experiments. For data analysis, paired t-test or one-way ANOVA with Dunnett’s or Bonferroni’s post test were applied. All P values resulted from two-sided statistical tests and statistical significance was set at *p < 0.05, **p < 0.01, and ***p < 0.001.

## Additional Information

**How to cite this article**: Liu, M. *et al*. Heme-mediated apoptosis and fusion damage in BeWo trophoblast cells. *Sci. Rep.* **6**, 36193; doi: 10.1038/srep36193 (2016).

**Publisher’s note**: Springer Nature remains neutral with regard to jurisdictional claims in published maps and institutional affiliations.

## Figures and Tables

**Table 1:** Primers for real-time polymerase chain reaction.

Primer set	Forward primer (5′ → 3′)	Reverse primer (5′ → 3′)
*Human LGALS13*	ATCAAAGGGACACCAATCCA	CAAAGTGCACTCGGAAACG
*Human CGB*	CGTCAACACCACCATCTGTG	ATGGACTCGAAGCGCACAT
*Human ERVWE1*	ACTGGGCTCCATGAGGTCT	TTCTGTGCTGAAGTTGTTCCA
*Human α-Fodrin*	CTGAAGAGCTGGAGAAATGGA	GCACTTCAGCTTCAAATGCTT
*Human GCM*	GACCTGCCATCTGTGACAAG	AGAAGTTGGTGACCGGGAAG
*Human ALPP*	CGCCAAGAACCTCATCATCT	ATGGCCAGGGGTATCTCAG
*Human Syncytin-2*	CCTTCACTAGCAGCCTACCG	GCTGTCCCTGGTGTTTCAGT
*Humane GAPDH*	GAAGGTGAAGGTCGGAGTC	GAAGATGGTGATGGGATTTC
